# Effect of laser Doppler flowmetry and occlusion time on outcome variability and mortality in rat middle cerebral artery occlusion: inconclusive results

**DOI:** 10.1186/s12868-018-0425-0

**Published:** 2018-04-19

**Authors:** Edvin Ingberg, Hua Dock, Elvar Theodorsson, Annette Theodorsson, Jakob O. Ström

**Affiliations:** 1 Department of Clinical Chemistry and Department of Clinical and Experimental Medicine, Linköping University, Linköping, Sweden; 2 Faculty of Medicine and Health, Örebro University, Örebro, Sweden; 3 Department of Neurosurgery and Department of Clinical and Experimental Medicine, Linköping University, Linköping, Sweden; 4 Department of Neurology, Faculty of Medicine and Health, Örebro University, Örebro, Sweden

**Keywords:** Rats, Middle cerebral artery occlusion, Ischemic stroke, Laser Doppler flowmetry, Mortality, Variability

## Abstract

**Background:**

Stroke is among the leading causes of death and disability. Although intense research efforts have provided promising treatment options in animals, most clinical trials in humans have failed and the therapeutic options are few. Several factors have been suggested to explain this translational difficulty, particularly concerning methodology and study design. Consistent infarcts and low mortality might be desirable in some, but not all, studies. Here, we aimed to investigate whether the use of laser Doppler flowmetry (LDF) and the occlusion time (60 vs. 45 min) affected outcome variability and mortality in a rat stroke model. Eighty ovariectomized female Wistar rats were subjected to ischemic stroke using intraluminal filament middle cerebral artery occlusion with or without LDF and with occlusion times of 45 or 60 min. Outcome was evaluated by triphenyl tetrazolium chloride staining of brain slices to measure infarct size and a modified sticky tape test.

**Results:**

Neither LDF nor occlusion times of 45 versus 60 min significantly affected mortality, outcome variability or outcome severity.

**Conclusions:**

Due to the unexpectedly high mortality and variability the statistical power was very low and thus the results were inconclusive.

**Electronic supplementary material:**

The online version of this article (10.1186/s12868-018-0425-0) contains supplementary material, which is available to authorized users.

## Background

Stroke is one of the leading causes of death in the world [1] and among those who survive, many suffer from chronic sequelae [2, 3]. In addition to the consequences for patients and relatives, the acute management and treatment for long-term complications also make stroke a major drain on health care funding [1, 4].

Intense research efforts have provided increased knowledge about stroke pathophysiology and a multitude of promising treatment strategies in animal models have been suggested [5, 6]. However, most clinical trials in humans have failed and hence the therapeutic options are still few [6]. Several factors have been suggested to at least in part explain this apparent translational difficulty, particularly regarding study quality [7]. One of the factors mentioned is underpowered studies, i.e. studies with too few animals per experimental group. Low statistical power (1 − β; β quantifies the type II error) has consequences regardless of whether the results turn out negative or positive [7, 8]. Statistical power is also related to outcome variability (high random variability decreases power) and mortality (high mortality decreased the power). The problem with low statistical power can be overcome by increasing the group sizes sufficiently, but to adopt such an approach in every study is problematic. Firstly, it is recommended to use as few animals as possible according to the ethical principle of “The three R’s” [9]. Secondly, working with a large number of animals is both costly and time consuming. Therefore, in some cases, a complementary solution is to optimize the rodent stroke model by minimizing outcome variability and mortality.

In rodent stroke experiments, the most popular method for inducing ischemia is intraluminal filament middle cerebral artery occlusion (MCAo) [10, 11]. This approach is relatively convenient and non-invasive, but the outcome variability and mortality can be substantial in many cases [7, 10, 11]. Two experimental factors that theoretically could have an impact on these parameters are occlusion time and the use of laser Doppler flowmetry (LDF), by affecting the extent of the lesion and ensuring correct placement of the filament, respectively. The potential influence of these factors has also been discussed by others [12, 13].

In the present study, we therefore aimed to investigate whether the use of LDF or 60 versus 45 min occlusion time affect outcome variability or mortality in a rat filament MCAo model.

## Results

**Fig. 1:**
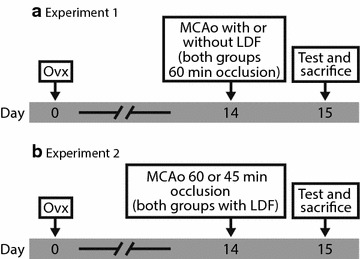
Study outline. The study comprised two separate experiments with similar design. The first part focused on LDF usage and the second on MCAo duration. *Ovx* ovariectomy, *MCAo* middle cerebral artery occlusion, *LDF* laser Doppler flowmetry

The study comprised two separate experiments with similar design. In experiment 1, the use of LDF was evaluated by allocating 40 ovariectomized female rats into two groups, with or without LDF, and 24 h after 60 min of MCAo the outcome was assessed by infarct size measurement and a modified sticky tape test. In experiment 2, the same procedures were undertaken, however, for this step the impact of occlusion time was investigated by comparing 40 animals subjected to 60 or 45 min of MCAo, respectively. For an overview, see Fig. 1.

### Outcome variability and severity

#### Infarct size

**Fig. 2:**
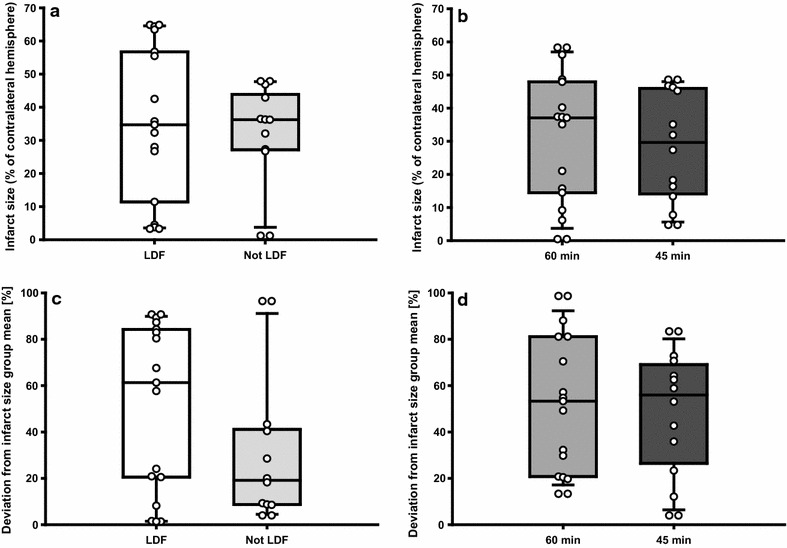
Infarct size and variability. No significant differences were observed when comparing LDF and not LDF, as well as 60 and 45 min, regarding infarct size (**a**, **b**) and infarct size variability (**c**, **d**). N = 15 (LDF), N = 10 (not LDF), N = 15 (60 min), N = 12 (45 min) after exclusion of animals that died. Circles represent the individual values, whiskers indicate the 90th and 10th percentiles

Neither experiment 1 nor experiment 2 showed any significant differences in infarct size between the groups (LDF vs. not LDF p = 0.935, Fig. 2a; 60 vs. 45 min p = 0.614, Fig. 2b). With mortality included, results were still non-significant (LDF vs. not LDF p = 0.189; 60 vs. 45 min p = 0.614). The same was true for infarct size variability, i.e. deviation of the individual infarct size values from the group mean, both without (LDF vs. not LDF p = 0.177, Fig. 2c; 60 vs. 45 min p = 0.943, Fig. 2d) and with dead animals included (LDF vs. not LDF p = 0.512; 60 vs. 45 min p = 0.461).

**Fig. 3:**
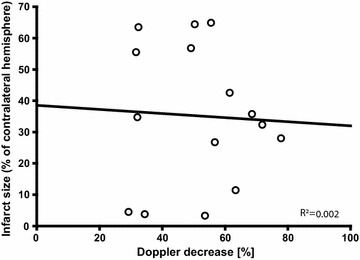
Correlation between LDF signal and infarct size. The correlation was very weak. Circles represent individual values from the LDF group in experiment 1, N = 15

The LDF signal decrease after filament insertion for the LDF group in experiment 1 was 51.2 ± 16.1% (mean ± SD), and the correlation with infarct size was very weak (R^2^=0.002; Fig. 3).

#### Modified sticky-tape test

**Fig. 4:**
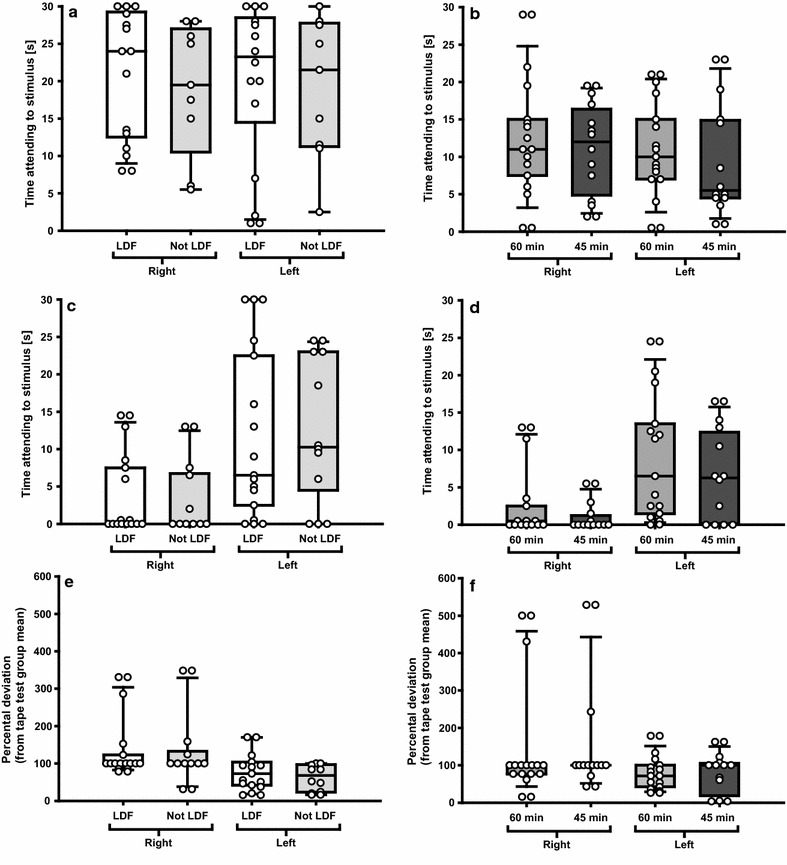
Modified sticky-tape test results. When comparing LDF and not LDF, as well as 60 and 45 min MCAo, no significant differences were found regarding outcome severity (measured as time attending to stimulus; **c**, **d**) or variability (**e**, **f**). The groups in experiment 1 and 2, respectively, were not significantly dissimilar at baseline (**a**, **b**). N = 15 (LDF), N = 10 (not LDF), N = 15 (60 min), N = 12 (45 min) in the tests before sacrifice, after exclusion of animals that died. Circles represent the individual values, whiskers indicate the 90th and 10th percentiles

For the sticky-tape test, neither the results for the groups in experiment 1 nor experiment 2 were diverging significantly from each other regarding test score at baseline (LDF vs. not LDF p = 0.983, Fig. 4a; 60 vs. 45 min p = 0.746, Fig. 4b), test score 24 h post-MCAo (LDF vs. not LDF p = 0.777, Fig. 4c; 60 vs. 45 min p = 0.381, Fig. 4d) or variability 24 h post-MCAo (LDF vs. not LDF p = 0.723, Fig. 4e; 60 vs. 45 min p = 0.427, Fig. 4f). When including mortality in the analyses, still no significances were found regarding test score 24 h post-MCAo (LDF vs. not LDF p = 0.121; 60 vs. 45 min p = 0.191) and variability 24 h post-MCAo (LDF vs. not LDF p = 0.127; 60 vs. 45 min p = 0.211).

For the right forepaw (impaired side), all groups differed significantly at 24 h compared to baseline test scores (LDF p = 0.008; not LDF p = 0.001; 60 min p = 0.001; 45 min p = 0.002). For the left forepaw (non-impaired side), two groups differed significantly (LDF p = 0.021; not LDF p = 0.006), while two did not (60 min p = 0.176; 45 min p = 0.061).

### Mortality

No significant differences in mortality rate were seen between the groups in experiment 1 or experiment 2 (LDF vs. not LDF p = 0.102; 60 vs. 45 min p = 0.409; Table 1).

**Table 1:** Mortality

Experiment	Group	Died during MCAo	Died post-MCAo
Experiment 1	LDF	0	5
	Not LDF	2	8
Experiment 2	60 min	1	4
	45 min	3	5

### Physiological monitoring

Data regarding oxygen saturation, respiratory rate and heart rate that were recorded during MCAo are presented in Table 2.

**Table 2:** Physiological parameters, measured continuously during surgery (average values presented)

Experiment	Group	Oxygen saturation, mean ± SD (%)	Heart rate, mean ± SD (bpm)	Respiratory rate, mean ± SD (brpm)
Experiment 1	LDF	95.6 ± 3.0	434.5 ± 37.8	52.5 ± 5.1
	Not LDF	97.9 ± 1.2	427.1 ± 47.6	51.4 ± 6.7
Experiment 2	60 min	95.8 ± 2.6	430.1 ± 33.1	55.4 ± 5.7
	45 min	96.5 ± 1.7	446.1 ± 29.9	56.7 ± 7.3

## Discussion

The objective of the current study was to investigate the effect of LDF usage and occlusion duration (60 vs. 45 min) on mortality and outcome variability in a rat filament MCAo model. Impact on outcome severity was also assessed. No significant differences were found. Due to the unexpectedly high mortality and variability the statistical power was very low and thus the results were inconclusive.

As described in the introduction, methodology and design of animal stroke studies have been intensively discussed over the last decade in the wake of repeated failures in bringing results from bench-to-bedside [5]. Low study quality, e.g. low statistical power, is believed to at least in part explain this discouraging track record. Although power is often discussed in relation to negative findings (the concept of publication bias and a study’s ability to detect an actual difference), from a bigger perspective it is of major importance also in studies where a treatment effect is found [8]. Several reviews have addressed these issues (e.g. [14]) and two meta-analyses (rats [11] and mice [10]) were performed in our lab to investigate method parameters’ impact on mortality and variability. High outcome variability will create noise in the statistical comparison and thus decrease power if sample sizes are not adjusted accordingly, and a high mortality rate requires larger group sizes to retain sufficient power. Two parameters, LDF usage and occlusion duration, were specifically targeted in the study described herein. They both have a bearing on this issue in that they theoretically could affect the consistency and the extent of the induced lesion, respectively, and therefore variability and mortality. Other factors that have been discussed or studied previously are e.g. strain [15–17], filament type [18] and blood glucose level [17, 19].

Mortality is an important issue in animal research. We mentioned above the ethical aspect and the indirect link to statistical power. A potential risk with excluding dead animals is also the scenario of a toxic substance that seems to decrease e.g. ischemic damage compared to placebo, only because all rats with large infarcts in the treatment group died. Mortality is often not even mentioned in rodent stroke studies [10, 11], which can distort interpretation of meta-analyses [20]. In the current study, mortality rate was higher than expected, 25–50% among the groups. Estimated power a priori was 0.83 [n = 20 per group, 10% mortality rate, 40 CV (Coefficient of variation) %, 40% difference between groups]. However, with our high mortality and variability, and considering that we used a non-parametric test, the power was around 0.30 (35% mortality rate, 65 CV%, 40% difference between groups), and even lower for a smaller difference between groups. This, in effect, renders the results inconclusive, as stated above. In an attempt to include mortality in the analyses we performed additional statistical tests to which animals that died after MCAo were added, considering death the most extreme outcome. Our mortality rate was high also compared to other similar studies; in our meta-analysis including 346 rat stroke studies the average mortality rate was around 15%. However, the range among the included studies was broad (0–60.4%), and a majority of the articles did not provide this information. Perhaps the average rate is not fully representative, it could be that authors with high mortality numbers were less keen on reporting this. Nonetheless, such high mortality as in the current study is ethically problematic and obstruct data interpretation. It is likely that insufficient surgical experience contributed to some extent, although since no sham group without MCAo was included, we cannot say for certain how much of the mortality was attributed to the ischemic damage and surgical complications, respectively. Further, analyses of potential causes of death among the animals, including infarct size measurement and checking for subarachnoid hemorrhage, would have been of value to refine the interpretation of the results.

The laser Doppler technique has been around for decades [21] and is widely used for filament MCAo models in rodents to monitor relative changes in cerebral perfusion [12, 22, 23]. The idea is to confirm correct and sufficient occlusion of the artery to induce consistent lesions among the experimental animals and/or to verify reperfusion. The sample volume seems to be only about 1 mm^3^ [23]. Although we did not find any effect of LDF usage at filament insertion, theoretically the use of LDF could reduce outcome heterogeneity in two different ways. Firstly, LDF-guided filament insertion could facilitate correct placement and in case of premature reperfusion during the intended occlusion period, the filament could be readjusted. Secondly, if an insufficient LDF signal drop is observed, that animal could be excluded from the study in order to avoid small or absent lesions. The second rationale, however, naturally assume a correlation between LDF signal decrease at occlusion and infarct size, which was not the case in the current study, nor in another study from our lab [24]. Several other researchers have also experienced this lack of correlation, both in rats and mice [25–28]. However, Riva et al. demonstrated that infarct size and functional deficit can be predicted with relevant probe positioning; they found that the border zone between the leptomeningeal branches of middle cerebral artery and anterior cerebral artery was superior to the lateral middle cerebral artery territory [29]. Further, there is a potential risk related to the use of LDF for exclusion; if the effect of a substance is mediated via changes in cerebral blood flow, this may be masked. A previous paper by Schmid-Elsaesser et al [12]. discussed that unnoticed filament dislocation may contribute to high variability but found that LDF was useful to identify and correct this. They also reported that LDF-guidance can prevent exaggerated filament insertion and thus reduce the risk of subarachnoid hemorrhage, which has further been corroborated by others [30, 31]. In a more recent paper, it was reported by Taninishi et al [32]. that LDF does seem to lower lesion size variation, but not neurologic outcome variation. Surgeons of varying experience participated in the Taninishi study and although the novice produced larger infarct with access to LDF, no effect was seen on outcome severity overall [32]. Our results were weak, but we did not observe any beneficial effect of LDF in contrast to previous findings. In the abovementioned studies, the LDF probe was fixated on top of the skull, against dura exposed by burr holes [12, 30–32]. Others reported similar positioning but thinned the skull, rather than exposed the dura [33]. Here we employed another approach with the probe placed without glue against non-thinned skull through the temporal muscle. Although this method has been used before [34–36], it is in some aspects different from the more conventional approach which could potentially have hampered the evaluation. Perhaps the signal was affected by the fact that the skull was not thinned, that the probe was not firmly fixated or that the positioning varied somewhat among the subjects—our probe placement was not based on coordinates in relation to bregma, but rather on external anatomical landmarks (eye and ear) as described in the methods section, possibly making the measurement less consistent. The LDF changes we observed were clear and instantaneous but relatively subtle (mean 51.2%) compared to what others reported with both the conventional (80% [31]) and current approach (80% [35]). This could indicate that our LDF monitoring was inadequately performed and perhaps produced a less detailed estimation of the perfusion. In addition, by using an unconventional technique the generalizability is decreased since the findings are not directly applicable for the majority of researchers working with the more common approach for LDF. Filament readjustment to counteract premature reperfusion during occlusion could not be evaluated since we did not keep the LDF during occlusion but rather let the animals be awake. Further, we did not put the LDF probe back on after re-anesthesia to monitor reperfusion at filament withdrawal. As mentioned above, mortality is a crude measurement and checking for subarachnoid hemorrhage among the dead animals could have provided more detailed information on whether LDF is useful to prevent this complication. LDF could possibly be a useful tool to ensure correct filament placement, and to adjust premature reperfusion if continuous monitoring throughout the procedure is practiced. However, based on the current findings, no firm conclusions can be drawn.

Although the choices regarding occlusion duration vary among researchers, some are more common. In our previous meta-analysis on methodological aspects in rat stroke studies [11], long transient occlusion (> 60 min; 56%) was more common than short transient (up to 60 min; 17.5%) and permanent (26.5%), and the median was 90 min. Since one of the goals of the current study was to achieve low mortality, shorter occlusion times were chosen (60 and 45 min). No significant differences were found when comparing 60 and 45 min occlusion and occlusion times longer or shorter than this were not included. However, logically at some point the results would diverge, with the two extremes being filament withdrawal immediately after insertion and permanent occlusion. Several previous studies in both rats and mice have showed that the longer the occlusion duration, the larger the infarcts [26, 37–39], indicating that an actual difference likely existed between our groups with 60 and 45 min occlusion, but that it was masked by high outcome variability (see the first paragraph of the “Discussion”). In a filament MCAo study by Mišir et al. [13], however focusing on diabetic rats, no significant difference in infarct size was found between 30, 45 and 60 min occlusion time, while 90 min increased it compared to the other groups and 20 min resulted in minimal or no infarcts. No statistical analyses were carried out regarding mortality rate or variability and conclusions should be made with caution, but some numerical data regarding this can be extracted from the article. Four out of 36 animals died, two in the 90 min group and two in the 60 min groups, while the shorter occlusion time groups had no mortality. Calculated coefficients of variation (CV; SD/mean), showed a pattern with higher variation in the groups with shorter occlusion (CV% 34.1, 21.5, 11.4, 15.1 for 30, 45, 60 and 90 min occlusion, respectively). Further, Encarnacion et al. [40] evaluated lesions 90 days after 60 or 75 min of filament MCAo in two different rat strains; for Sprague Dawley rats longer occlusion duration caused larger infarcts, which was not the case for Wistar rats (used also in the present study). No proper comparisons regarding mortality and variability were described in that paper either, but the mortality numbers were somewhat higher and CV somewhat lower with 75 min occlusion. Concerning ischemia duration and outcome severity, it is worth keeping in mind that depending on the objective of the study the goal might range from minimal striatal ischemia to massive hemispheric infarct. Therefore only one desired lesion type cannot be defined.

In addition to what has been mentioned previously in the discussion, a few other weaknesses of the current study should be addressed. Only ovariectomized female rats were used since one of the purposes of this study was to implement the results in our research on rodent menopause models. In such studies, ovariectomized female animals are often used, and treatment (for example hormone replacement therapy) is administered to mimic the clinical situation. Including males would have increased the generalizability of the results since males are most often used in stroke studies [11]. It also would have been an option to have non-ovariectomized females, but in that case it would have been necessary to consider stage in the estrous cycle since this can affect stroke outcome [41]. Further, although outcome evaluation only after 24 h is the most common approach [24], it is known that the infarct changes also after this [42, 43]. Long-term follow-up with multiple evaluation time points would have provided a more detailed assessment. Moreover, duration of exposure to anesthesia was not recorded for each animal and thus it was not explored how this might have contributed to outcome severity and variability; Isoflurane has been shown to improve neurological outcome after MCAo [44, 45]. Worth mentioning is also the important drawback that the sticky-tape test was not performed in a completely blinded fashion (see “Methods”).

## Conclusions

Due to the unexpectedly high mortality and variability the statistical power was very low and thus the results of the present study were inconclusive. We attempted to statistically assess the value of LDF or whether 60 or 45 min of filament-induced middle cerebral artery occlusion are to be recommended if the goal is to minimize mortality and variability, but no firm conclusions can be drawn. Decreasing mortality and variability can provide sufficient statistical power with fewer animals, which could be of particular interest for studies of more explorative and hypothesis-generating nature. In confirmatory pre-clinical studies, aiming to evaluate e.g. a drug before moving on to clinical trial, low mortality and very consistent lesions might not be always the goal. Brain infarcts in humans are heterogeneous, and to examine the robustness of animal results this diversity should be considered and mimicked. In such cases, larger group sizes are needed to maintain power.

## Methods

### Study outline

The study comprised two separate experiments with similar design. All rats were ovariectomized 2 weeks before each experiment. In experiment 1, the use of LDF was evaluated by randomly (random.org) allocating 40 animals into two groups (20 per group), with or without LDF, and 24 h after 60 min of MCAo the outcome was assessed by infarct size measurement and a modified sticky tape test. In experiment 2, the same procedures were undertaken, however, for this step the impact of occlusion time was investigated by comparing 40 animals randomly divided in two groups (20 per group), subjected to 60 or 45 min of MCAo, respectively. For an overview, see Fig. 1. One experimenter performed the stroke surgeries and another experimenter performed the behavioral testing, sacrifices, and infarct size analyses. The infarct sizes were assessed in a randomized and blinded fashion, but the behavioral testing cannot be considered completely blinded; behavioral testing and MCAo procedures were performed in the same room and the person performing the behavioral testing could have unintentionally picked up what groups the stroked rats from the previous day belonged to.

### Animals

A total of 80 female Wistar rats (Taconic Europe, Ry, Denmark; 14 weeks, mean ± SD 296 ± 22 g) were pre-MCAo housed two in each cage with access to nesting material, wooden chew sticks and cardboard cylinders and solitarily without enrichment post-MCAo. The animals were kept in 22 °C on a 12-h light/dark cycle and with standard rodent chow and water provided *ad libitum.*

### Surgical procedures

Ovariectomies were performed via the dorsal route [46]. Following 14 days of washout, left-sided MCAo was performed on each animal based on the original descriptions by Koizumi [47] and Longa [48]. A 2 cm cervical midline incision was made and the common (CCA), internal (ICA) and external (ECA) carotid arteries were freed from surrounding tissue. After ligation of the CCA and ECA, the ICA was temporarily clipped (8 mm artery clip, Rebstock Instruments Gmbh, Dürbheim, Germany). Thereafter, a 30 mm silicone coated 4–0 nylon suture (403756, Doccol, Redlands, CA, USA) was inserted in the CCA and advanced up the ICA. For the LDF group in experiment 1 and both groups in experiment 2, LDF of the left hemisphere was used (moorVMS-LDF, Moor Instruments, Axminster, Devon, UK), using a previously described approach [34–36]. After shaving and cleaning of the area, a 0.5 cm incision was made between the left eye and ear and the connective tissue and temporal muscle was dissected apart while avoiding tearing or cutting nerves. The probe was then positioned against the non-thinned skull through the temporal muscle and secured externally with adhesive tape. Baseline LDF values were around 200 PU and the change in Doppler signal at filament insertion was recorded (percent decrease calculated from 30 s before and 30 s after filament insertion). For the rats in the group without LDF, the filament was inserted until a slight resistance was felt, approximately 18–20 mm. After 60 or 45 min of occlusion (60 min for experiment 1, and 60 or 45 min for experiment 2), the filament was withdrawn and the ICA was permanently ligated. The rats were awake during the occlusion. Postoperatively, the rats recovered in heated cages (30 °C) for 15 min and water-soaked food pellets were provided in a petri dish on the cage floor to facilitate eating.

During ovariectomy and MCAo, the rats were anesthetized with Isoflurane (Forene^®^, Abbott Scandinavia AB, Solna, Sweden), 4.5% for induction and 1.5% for maintenance delivered in a 30/70 mixture of O_2_/N_2_O. As pain relief, 5 mg/kg bodyweight subcutaneous carprofen (462986, Rimadyl Vet, Pfizer ApS, Ballerup, Denmark) was used for ovariectomy and 1.25 mg/kg bodyweight subcutaneous bupivacaine (Marcain, AstraZeneca, Södertälje, Sweden) for MCAo. Before MCAo, 5 mL saline was given as fluid replenishment. Oxygen saturation, respiratory rate and heart rate were recorded by pulse oximetry (SLS-MO-00404, MouseOx, Allison Park, PA, USA). Body temperature during surgeries was maintained at 37 °C with a heating pad coupled to a rectal thermometer feedback system (50-7061, Harvard Apparatus, Holliston, MA, USA).

### Measurement procedures

#### Modified sticky-tape test

The modified sticky-tape test was performed in a manner similar to the procedure described by Sughrue et al. [49]. This modified approach was chosen since it has been shown to be superior to the conventional test in that it requires less pre-training and better differentiate between the affected and non-affected limb [49]. Briefly, a 30 mm segment of green autoclave tape was wrapped around one forepaw at a time. The rat was then placed in a transparent cage (36 × 19.5 × 18.5 cm) and during 30 s, time spent attempting to remove the tape sleeve either by scratching with the other paw or biting, was recorded. On the few occasions when the rat managed to remove the tape sleeve, the time attending the stimulus was set to the maximum 30 s. The test was performed pre-MCAo (baseline) and 24 h post-MCAo and the average of two testing sessions per rat on each occasion was used. The results for the right forepaw (impaired side; contralateral to the ischemic hemisphere) was used in the statistical comparisons.

#### Lesion measurement

**Fig. 5:**
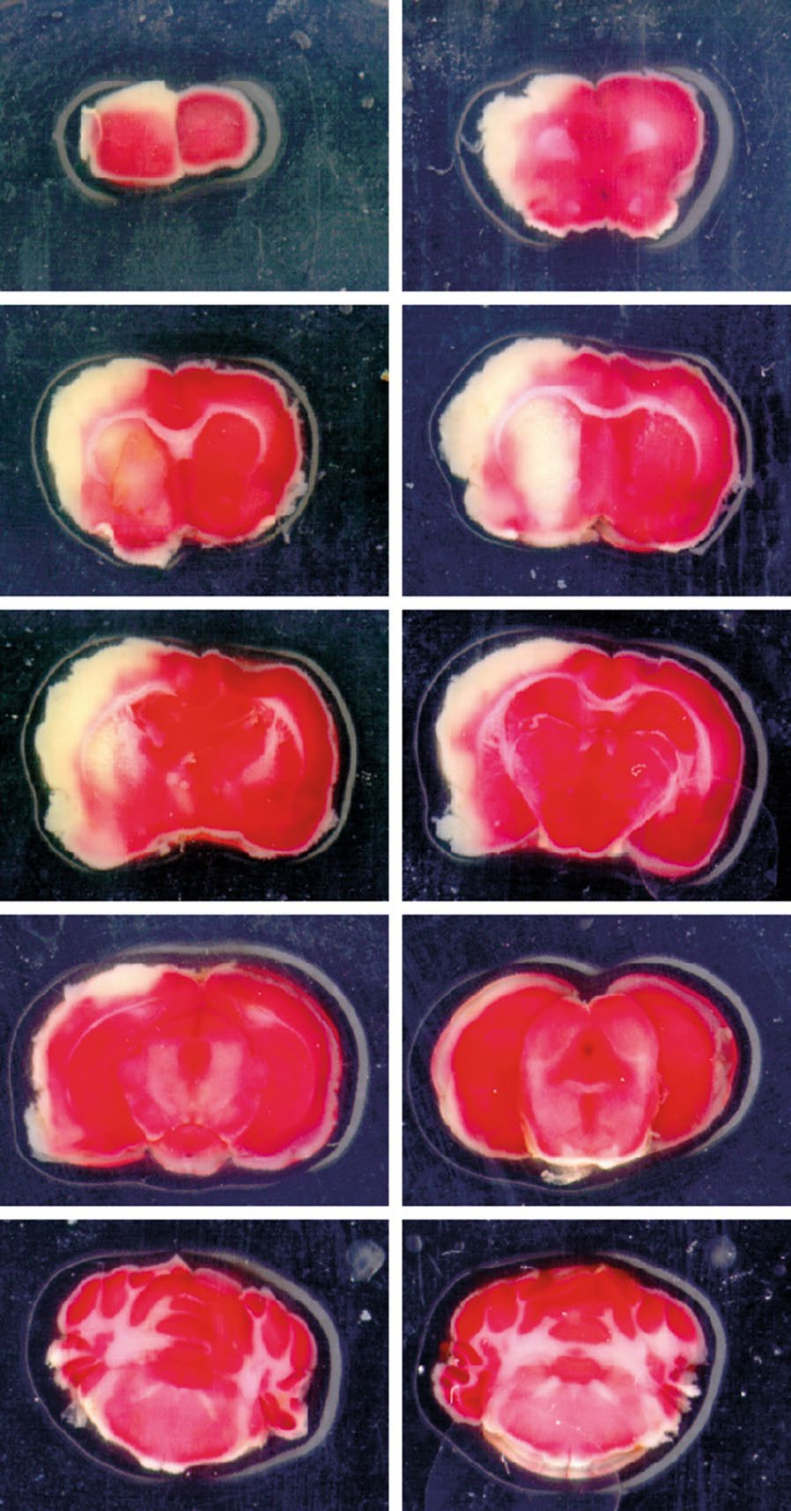
Example of scanned brain slices from a rat subjected to 60 min of middle cerebral artery occlusion. The brains were sliced in 2 mm slices that were soaked in 2% triphenyl tetrazolium chloride solution (TTC) for 15 min and subsequently scanned

24 h after MCAo, the rat was anesthetized with Isoflurane (Forene^®^, Abbott Scandinavia AB, Solna, Sweden), 4.5% delivered in a 30/70 mixture of O_2_/N_2_O, and decapitated using a rodent guillotine. The brain was dissected out, cooled in ice water for 5 min and sliced in 2 mm slices using a rat brain matrix (RBM-4000, ASI Instrument Inc., USA). The slices were subsequently soaked in 2% triphenyl tetrazolium chloride solution (TTC; Sigma-Aldrich Sweden AB, CAS# 298-96-4, Stockholm, Sweden) at 37 °C for 15 min and scanned (ScanJet 2c, Hewlett-Packard, Palo Alto, CA, USA). An example of TTC stained brain slices are provided in Fig. 5. Infarcts were measured at large according to the method described by Goldlust et al.’s [50], with an automatic 40% green spectrum threshold (SigmaScan Pro 5, Systat Software Inc, San Jose, CA, USA). Edema correction was performed according to the following formula:

$$ {\text{Corrected}}\;{\text{infarct}}\;{\text{volume}},\;{\text{as}}\;{\text{part}}\;{\text{of}}\;{\text{one}}\;{\text{hemisphere}} = \left[ {\text{crude}}\;{\text{infarct}}\;{\text{area}} \times  \left[{\text{contralateral}}\;{\text{hemisphere/ipsilateral}}\;{\text{hemisphere}}\right]\right] / {\text{contralateral}}\;{\text{hemisphere}} $$

### Statistics

In order to contrast outcome variability between groups, percent deviation from group mean was calculated for each animal regarding infarct size and sticky-tape test results. This value was subsequently used in the statistical calculations. For example, if the mean infarct size for a group was 0.35 and a certain rat had a value of 0.5, the percent deviation from mean would be (0.5 − 0.35)/0.35 = 0.43, i.e. 43% deviation. The Mann Whitney U test was used for comparisons of variability, infarct sizes and sticky-tape test outcome (time attending the stimulus), between LDF versus not LDF and between 60 versus 45 min occlusion. A non-parametric test was chosen based on lack of normality. In an effort to include mortality in the analyses, additional Mann Whitney U tests were performed to which animals that died were added. For outcome severity, dead animals were assigned the worst possible outcome. For variability analyses, dead animals were considered to have the most the extreme deviation from the group mean value. Such analyses were possible since non-parametric statistics (using ranks) were used. Chi squared test was used for comparison of mortality rate for LDF vs not LDF and 60 versus 45 min occlusion. Sticky-tape test scores for all groups at 24 h and at baseline were compared for the right (impaired) and left (non-impaired) forepaws, respectively, with Wilcoxon signed-rank test. Data are presented as median and interquartile range, unless stated otherwise. A significance level of 0.05 was used.

#### Protocol violations

A total of four animals were too stressed to perform baseline sticky-tape testing, and could therefore not contribute to these results. Of the remaining, 18 animals went through only one baseline testing session for the same reason. Four animals were very affected by the stroke and went through only one testing session 24 h post-MCAo. Due to technical issues, for ten of the surviving animals Doppler data are missing.

## Additional file

**Additional file 1.** Data on which “Effect of laser Doppler flowmetry and occlusion time on outcome variability and mortality in rat middle cerebral artery occlusion: inconclusive results” was based on.

## Abbreviations

MCAo: middle cerebral artery occlusion
LDF: laser Doppler flowmetry
CV: coefficient of variation
CCA: common carotid artery
ICA: internal carotid artery
ECA: external carotid artery
TTC: triphenyl tetrazolium chloride
